# Development of Diallyl Phthalate-Filled Ceramic Shell Self-Healing Capsules for High-Temperature Polymer Composites

**DOI:** 10.3390/polym17121621

**Published:** 2025-06-11

**Authors:** Murat Yazıcı, Aycan Karaman, Eslem Şahin, Gönenç Duran

**Affiliations:** 1 Applied Mechanics and Advanced Materials Research Group (AMAMRG) Laboratory, Automotive Engineering Department, Engineering Faculty, Bursa Uludağ University, Bursa 16059, Türkiye; aycankaraman@uludag.edu.tr (A.K.); eslem.sahin@mudanya.edu.tr (E.Ş.); gonenc.duran@mudanya.edu.tr (G.D.); 2 Automotive Technology Program, Department of Motor Vehicles and Transportation, Vocational School, Mudanya University, Çağrışan Campus, Bursa 16960, Türkiye

**Keywords:** self-healing, ceramic capsules, diallyl phthalate (DAP) resin, peroxide, epoxy resin

## Abstract

In this study, a production method for ceramic shell macrocapsules and a high-temperature-resistant, polymer agent-based self-healing system was developed. Two types of macrocapsules were created by filling hollow ceramic capsules with high-temperature-resistant diallyl phthalate (DAP) resin, known for its thermal stability, and a peroxide-based curing agent. These capsules were incorporated into epoxy and DAP matrix materials to develop polymer composite materials with self-healing properties The macrocapsules were produced by coating polystyrene (PS) sacrificial foam beads with raw ceramic slurry, followed by sintering to convert the liquid phase into a solid ceramic shell. Moreover, FTIR, TGA/DTA, and DSC analyses were performed. According to the thermal analysis results, DAP resin can effectively function as a healing agent up to approximately 340 °C. In addition, quasi-static compression tests were applied to composite specimens. After the first cycle, up to 69% healing efficiency was obtained in the epoxy matrix composite and 63.5% in the DAP matrix composite. Upon reloading, the second-cycle performance measurements showed healing efficiencies of 56% for the DAP matrix composite and 58% for the epoxy matrix composite.

## 1. Introduction

Self-healing materials are a new class of materials designed with the principle of biomimetics and are resistant to mechanical damage. These materials could self-repair microcracks and superficial abrasions thanks to low molecular weight repair agents, which usually act in polymer networks. This phenomenon offers significant advantages in terms of sustainability and cost-effectiveness by extending the service life of materials [1,2,3,4].

The healing process begins with activating the repair agents placed in the areas where micro- and macrocracks may occur in the material. The agents used can be of various types, such as microcapsules [5,6,7,8], vascular systems [9,10] or polymer networks [11,12,13]. The ability of the material to sense damage and repair itself without the need for any external intervention is vital to quickly and effectively repair unexpected damage, especially in bridges, airplanes, and other critical applications. It provides significant advantages, especially when access is difficult or cannot be kept under constant surveillance [14,15,16,17]. However, there are some disadvantages of these materials, including their high production cost compared to traditional materials, limited repair capacity, and the fact that repair can take a long time, depending on the size of the damage. In addition, changes in the physical and chemical properties of the material due to environmental conditions can lead to a loss of performance [18].

Self-healing materials include many types, such as polymers, elastomers, metals, ceramics, and cement. Systems that allow the combination of different material classes make it possible to produce sustainable materials. Low-viscosity self-healing agents leave microcracks on damaged areas for subsequent curing and filling. The self-healing materials approach varies in healing efficiency depending on the location of the damage, the type of resin, and the effect of the operational environment [19,20].

Thermoset materials commonly used in structural applications have thermal stability and rigidity that thermoplastics lack. Thermosets, which are cross-linked molecules, exhibit distinctive properties with their varied chemistry and molecular structures. DAP is an ester of phthalic acid containing two allyl groups. It stands out among thermosetting resins by offering exceptional dimensional stability, insulation, low water absorption and excellent electrical properties. DAP is a plastic molding compound widely used in components such as high voltage insulators and distributor covers, particularly in the automotive and electrical industries. It is also preferred in sectors that require high performance, such as aviation and space. However, molding at high temperatures and compressions, also presents disadvantages, including long curing times and incurred costs [21,22]. Today, studies such as the modification of DAP with nanomaterials are aimed at further improving the properties of this material and reducing its cost [23]. Synthetic phthalates are particularly effective in providing low-temperature flexibility and functioning as emollients. Research has demonstrated that the particle size of DAP plays a critical role in enhancing the mechanical properties of materials. Reducing the particle size to a nanoscale level is assumed to achieve significant efficiency [23,24]. In the studies on the lap-shear adhesion strength of DAP resin modified with allyl ester compounds, the adhesion of different materials was tested. As a result of these studies, it has been stated that the interface interaction between the resin and the material has increased [25]. Moreover, it is shown that the deposition of polymerized DAP layers resulted in improved interfacial shear strength and impact properties of the composite materials, especially non-polar polyolefin matrices such as polypropylene (PP) [26]. These interfacial interactions contribute to enhancing the long-term performance of composites by improving their structural integrity and durability [27].

On the other hand, ceramic materials are used in mechanical parts, cutting tools and coating materials with advantages such as high strength, high thermal shock resistance, low thermal expansion coefficient, high-temperature resistance, high creep resistance, low density and high corrosion resistance. However, due to their high brittleness, ceramic materials are very sensitive to micro-defects and can be easily broken due to external factors [28,29,30]. Thanks to its high temperature and chemical resistance properties, ceramic capsules can safely store the active agents filled inside, breaking and releasing them in case of damage. Although these capsules are usually fragile, they effectively preserve their active substances due to their chemical stability and low permeability [31].

These properties of DAP and ceramic materials can be successfully combined in self-healing composite systems. The flexibility of DAP and the chemical resistance of ceramic capsules can combine to create materials that have both high mechanical performance and the ability to repair themselves [32]. When the ceramic capsules are integrated into the DAP resin, the repairing agent is released by breaking the ceramic capsules during cracks or damages that may occur, and these agents fill the crack and allow the material to regain its former strength. Such integration offers great potential for smart materials developed specifically for use in areas that require high strength and longevity, such as automotive and aerospace.

Such composites, obtained with a heterogeneous structure, are prone to various damage modes such as intralaminar and translaminar fracture, fiber breakage and matrix fracture. These modes of damage are determined by the interactions between the interface (the boundary surface between the shell and the matrix) between the capsule and the matrix and the properties of the different phases within the material. Theoretically, damage repair (autonomously or by external intervention) can restore the matrix properties that have been degraded due to these damage modes and thus extend the life of the composite.

Mechanical tests such as quasi static bending, compression, torsion, and impact are commonly used to determine the percentage healing of the selected mechanical property to measure the quality of the repair function. Before and after self-healing, the properties of the test specimens, such as hardness and strength, are determined. A dimensionless value is then calculated, expressing the healing percentage of the relevant mechanical property. The healing efficiency is calculated as in Equations (1) and (2) [2,3,33,34,35].

Equation (1) defines the absolute improvement efficiency, $\eta \;\left(\%\right)$, representing the percentage improvement of a mechanical property compared to the original (undamaged) material after the self-healing process. It is a simple and widely used equation in the literature to evaluate how effectively a material recovers its function after damage.

(1) $$\eta \;\left(\%\right)=100\left[\frac{Healed\;property}{Pristine\;property}\right]$$

Equation (2) represents a damage-based remediation efficiency expressed as $\eta^{\prime }\;$(%), where the improvement due to remediation is calculated relative to the deterioration caused by the damage:

(2) $$\eta^{\prime }\;\left(\%\right)=100\left[\frac{Healed\;property-damaged\;property}{Pristine\;property-damaged\;property}\right]$$

In this second formulation, the damaged property refers to the measured value of the mechanical property after controlled damage has been applied but before any healing process has taken place. This is typically performed by first inducing mechanical damage, then comparing it with the values obtained after healing and in the pristine state.

Ceramic macrocapsules have advantages such as high temperature resistance, chemical stability, and long-term preservation of the repairing agent in its content. In addition, ceramic capsules are highly resistant to external environmental conditions and have a non-reactive structure [36]. Ceramic macrocapsules can resist mechanical stresses during material manufacturing and capsule integration with good compression strength properties [37]. However, they are relatively brittle. Therefore, it reduces the healing agent leaking reaction time around stress cracks in the self-healable materials obtained by incorporating these capsules.

In this study, the insides of these capsules, which have stability in the matrix during the production process, were filled with DAP resin, which is used as a healing element. The healing performance of the material after the damage was examined. According to the literature review conducted by the authors, no self-healing study was found among the accessible open sources using DAP resin-filled ceramic capsules presented in this article. This is planned as a pioneering study for high-temperature polymer composites with self-healing properties.

## 2. Material and Method

Ceramic casting clay (Refsan/Türkiye) containing 55% silicon dioxide (SiO_2_) and 17% Alumina (Al_2_O_3_), thermoset DAP resin from plasticizer class, peroxide-based curer, and Ecomar R15 epoxy A-B resins were used in the study.

**Figure 1:**
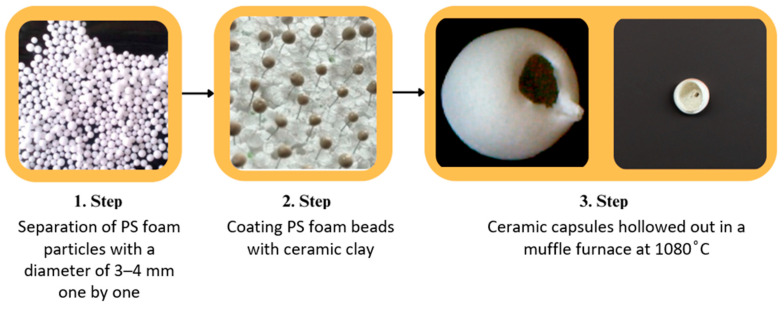
Empty capsule production steps.

First, polystyrene foam particles were coated with ceramic clay to produce capsules. In two steps, PS foam beads coated with ceramic clay were left to pre-dry at 60 °C. The pre-dried capsules were coated with ceramic clay for a second time. It was then sintered at 1040 °C for 4 h. The PS foam coated in this step also melted and formed a hollow capsule (Figure 1). As a result of the first coating, a thin-walled fragile shell structure was obtained. A more rigid shell structure was formed when the second coating was made.

**Figure 2:**
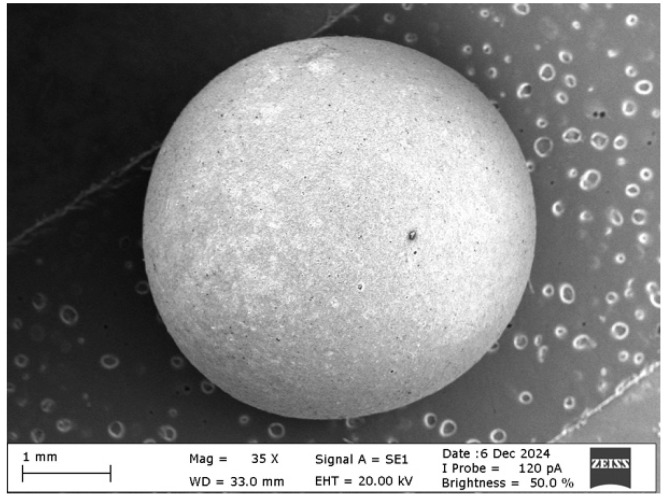
SEM image of a single capsule viewed at 35× magnification.

**Figure 3:**
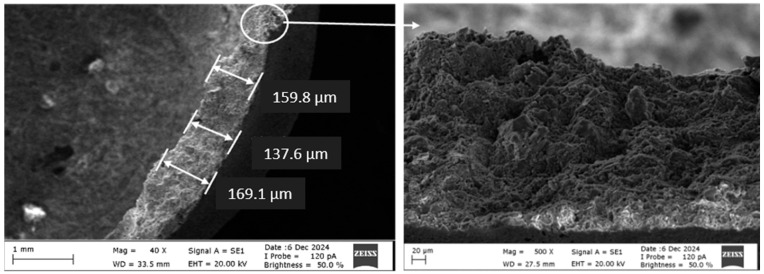
Capsule wall thickness and SEM image.

The diameter of the capsules varies on average around 4.5 ± 0.2 mm (Figure 2), while the thickness of the shell structure varies to an average of 150 ± 18 μm (Figure 3).

**Figure 4:**
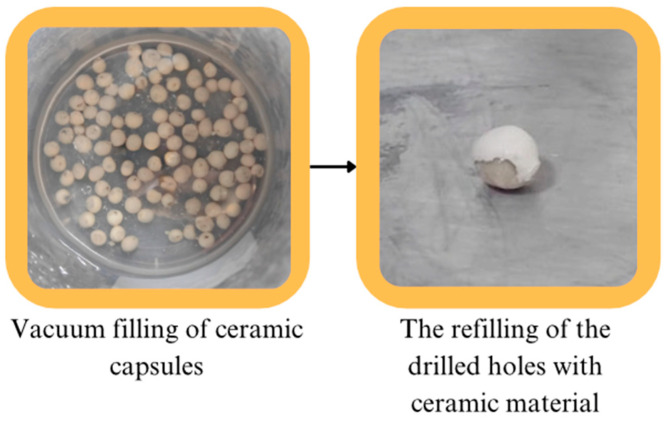
Obtaining filled capsules.

The hollow capsules obtained were impregnated separately with DAP resin and peroxide-based curer, which are healing agents, by vacuum method. The filling cavities were closed again with ceramic clay and made ready to be placed in the matrix (Figure 4).

Since a peroxide-based initiator is used for curing, these initiators are usually added during application. This results in a two-component reaction from a one-component system.

FTIR (Fourier Transform Infrared Spectroscopy), TGA (Thermogravimetric Analysis), and DSC (Differential Scanning Calorimetry) tests were performed to characterize the DAP resin. These methods have been used to understand the chemical structure, thermal stability, and thermal transitions of DAP resin used as a healing agent.

### 2.1. FTIR Test

DAP resin, curing agent, and post-curing structures supplied as healing agent were examined by FTIR test. The test was carried out on the Shimadzu IRTracer—100 branded device.

FTIR analysis was used to determine the presence of functional groups in the chemical structure of DAP resin and the placement of these groups. Prominent peaks in the spectrum provided information on the chemical structure of the resin. For example, the peaks of C=O and C-O bonds revealed the characteristic features of the DAP structure. These results confirm that the resin has the correct structure and can be used as a healing agent.

### 2.2. TGA/DTA Test

TGA test of the structure formed because of curing was performed. The test was carried out on the Shimadzu DTG-60H branded device.

TGA analysis was performed to determine the thermal stability and degradation temperatures of the DAP resin. The TGA curves showed the mass loss of the material at certain temperatures and thus the thermal strength range of the resin was determined. This analysis is important to assess whether the resin is suitable for the temperatures to be used in the healing process. The results obtained showed that the DAP resin has thermal stability over a certain temperature range.

DTA allows physical or chemical changes in the material to be associated with energy. When used with TGA (Thermogravimetric Analysis), it allows simultaneous analysis of mass loss and thermal phenomena.

### 2.3. DSC Test

A DSC test of the structure formed because of curing was performed. The test was carried out on the DSC TA Instrument—DSC 25 branded device to analyze the thermal properties of specimens in argon environment with heating and cooling rates of 5 °C/min.

DSC analysis was performed to determine the thermal transitions (e.g., glass transition temperature, melting point) of the DAP resin. This analysis is critical to understanding the compatibility of the resin with the polymer matrix and the behavior it will exhibit during the healing process. The DSC results revealed the thermal transition points of the DAP resin and the effects of these transitions on the healing process. These transitions determined whether the resin could be properly integrated into the material.

### 2.4. Compression Test

**Figure 5:**
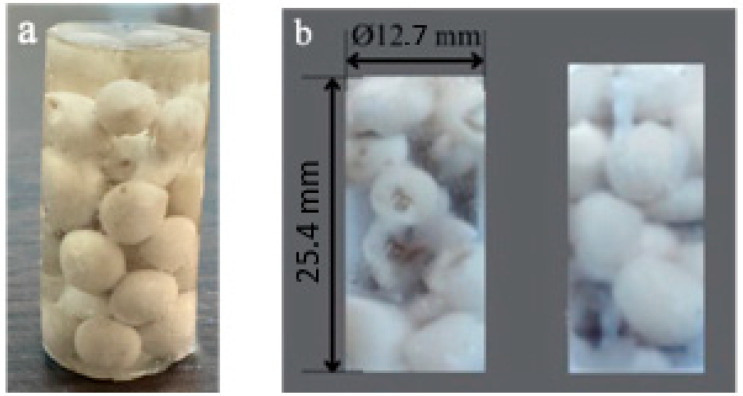
(**a**) compression test specimen, (**b**) zoomed view and dimensions of the compression test specimen.

Compression specimens containing DAP-filled ceramic capsules in a matrix of Epoxy were prepared according to ASTM D695 standard (Figure 5). Approximately 20 capsules were placed in the cylindrical compression specimen. Of these, 16 were DAP-filled and 4 were peroxide-filled ceramic capsules with curing material. Five specimens were tested for each test.

**Figure 6:**
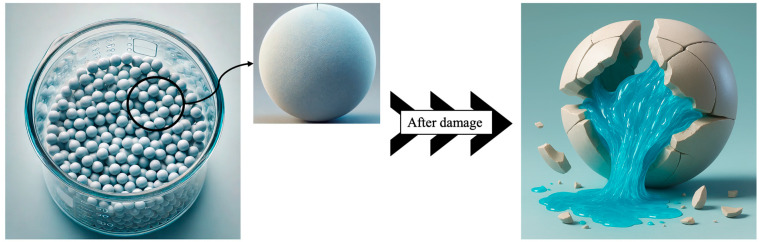
Image of a possible fracture after compression damage of the capsules produced.

A compression test was applied to the specimens obtained using the matrix material as epoxy in the compression specimens produced by the same standards. The resistance force of single capsules with a diameter of 4 ± 0.5 mm to compressive force was tested at a speed of 2 mm/min. Figure 6 shows the fracture of a single capsule.

## 3. Results

### 3.1. FTIR Test Results

**Figure 7:**
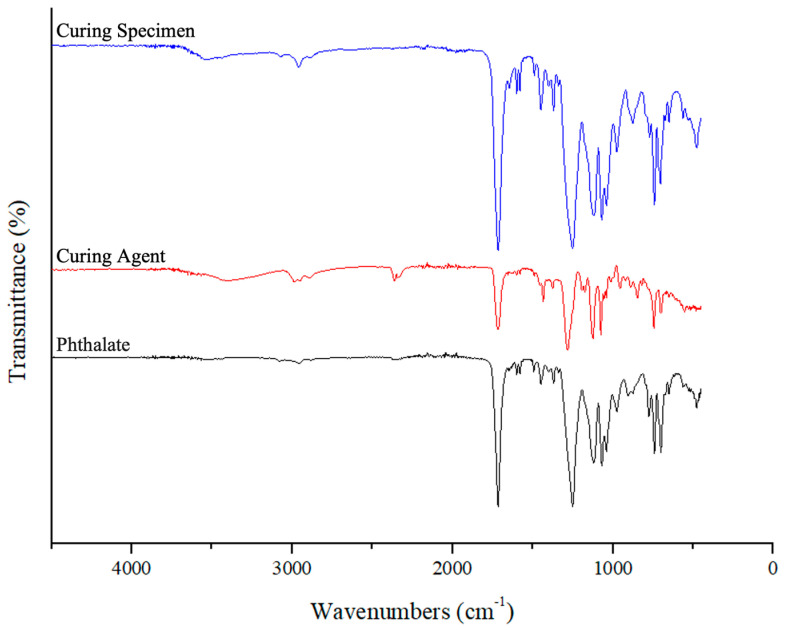
FTIR results of resins.

The FTIR results of the DAP resin and curer supplied as healing agents are given as shown in Figure 7.

Its spectrum, which belongs to DAP resin, has a distinct carbonyl peak in the region of 1700 cm^−1^. There are C-O and C-H vibrations in the 1200 cm^−1^ and 1000 cm^−1^ regions. In the graph of the peroxide-based curing agent, alkene C-H vibrations are in the region of 3000 cm^−1^. It has a carbonyl peak in the region of 1700 cm^−1^. C-O and C-H vibrations are present in the 1200 cm^−1^ and 1000 cm^−1^ regions. The spectrum of peroxide-cured resin has a carbonyl peak in the region of 1700 cm^−1^. C-H pulsations are present in the 1400 cm^−1^ and 1000 cm^−1^ regions. These spectra, which show the properties of both materials, show the cured structure.

### 3.2. TGA/DTA Test Results

**Figure 8:**
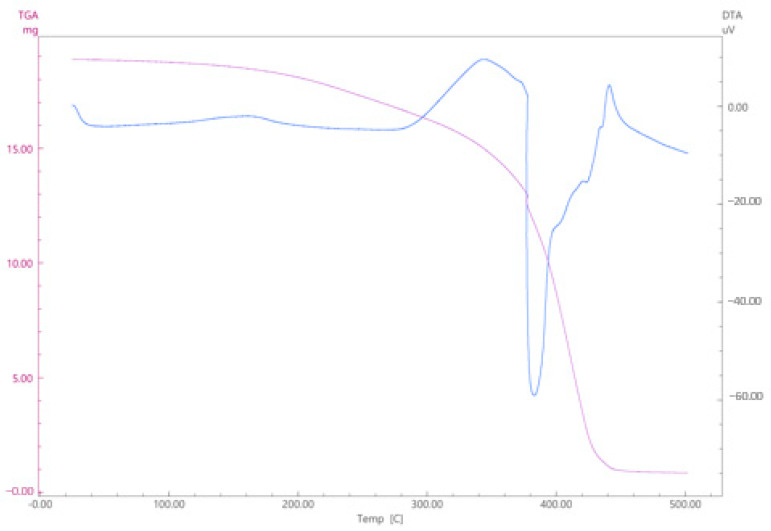
TGA/DTA test results.

In the TGA graph (Figure 8), which gives the temperature-mass loss, no significant change in mass was observed at the initial temperature increase (0–300 °C). This region has a stable structure. After 300 °C, a rapid mass loss was observed. Especially around 400 °C, there is a great mass loss. This mass loss suggests that DAP resin has begun to decompose, and volatile components are separated. After 400 °C, almost all the masses were lost, indicating complete thermal degradation.

In the DTA test in the same graph, the *y*-axis shows the thermal changes. Between 300 and 400 °C, there is a significant endothermic peak. With the breaking of chemical bonds, an increase in energy is observed during degradation. After 400 °C, an exothermic reaction appears. This may indicate a combustion or carbonization reaction.

As a result, thermal degradation of DAP occurs in the 300–400 °C range. The endothermic peaks seen in the DTA curve confirm that the structure has degraded, and its thermal stability ends in this temperature range. The rapid mass loss in the TGA curve indicates that volatile products are separating, and the structure is completely disintegrating. These analyses indicate that DAP loses its thermal stability at temperatures above 350 °C, and its use may be limited in this temperature range. However, for practical usage of self-healing applications such as crack filling in high-temperature subjected or high-temperature processing polymer matrix composite materials, the temperature range of 320–340 °C can be considered as the upper limit.

### 3.3. DSC Test Results

**Figure 9:**
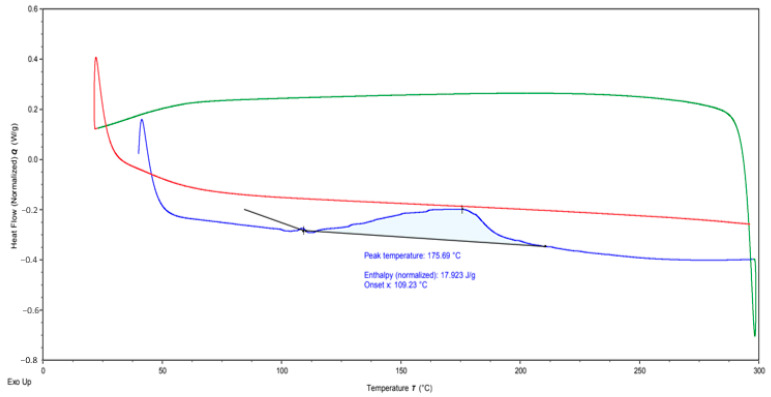
DSC test results.

The green curve shown in Figure 9 shows endothermic reactions. This curve shows that the material takes up heat. It is usually associated with melting, glass transition temperature (Tg) or other endothermic reactions. The graph indicates a prominent peak at 175.69 °C, signifying a phase change in the material at this temperature. The red curve is exothermic responses. This curve shows that the material gives off heat. This type of reaction is usually associated with exothermic reactions such as polymerization or crystallization. The fact that the curve here is negative indicates that heat is being released, and a reaction is taking place. The blue curve, on the other hand, is related to the enthalpy of the material. The value of the enthalpy is given as 17.923 J/g. In addition, the starting temperature (onset) is specified as 109.23 °C. This is indicated as the temperature at which the first thermal change in the material begins.

This analysis is useful to understand the thermal properties of the material and to learn about its possible uses. It provides an important reference point when assessing the thermal stability and reactions of the material.

### 3.4. Compression Test Results

The effect of different matrix materials on capsule mechanical behavior was investigated. In this context, compression tests were performed, and epoxy and DAP resin matrices were evaluated.

**Figure 10:**
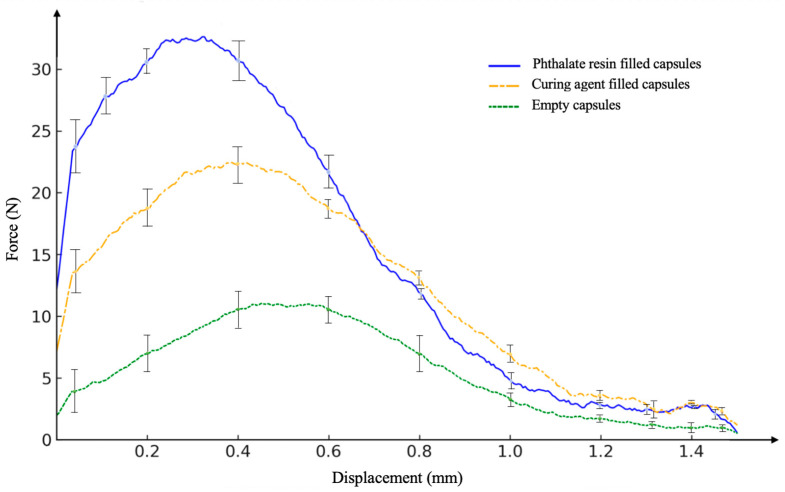
Single capsule compression test results.

Figure 10 shows force-displacement results of compression experiments for ceramic capsules which are DAP-filled, curing agent-filled, and empty capsules. The highest strength value has been obtained in a DAP-filled capsule. As expected, the filling material increases the mechanical strength of the capsule. The curve reaches the highest maximum force value relative to the other capsules (35 ± 0.98 N). This indicates that the filler supports the microcapsule’s shell, allowing it to carry more load. The sudden drop of the curve after the maximum point of force indicates the fracture behavior of the capsule. The empty capsule shows the lowest mechanical performance. The lack of internal filling material limits the load-bearing capacity of the structure. The maximum strength value is around 10 ± 0.63 N. This demonstrates the critical impact of the filling material on capsule mechanical performance. In the empty capsule, ductile behavior can be observed. The curve gradually decreases and there is no sudden breakout.

The capsule filled with a peroxide-based curing agent exhibits moderate strength. Although it shows a lower strength than the resin-filled capsule, it provides a significant mechanical gain compared to the empty capsule. The maximum strength value is around 20 ± 0.86 N. This value indicates that the filling agent imparts some load-bearing capacity to the capsule. After the maximum strength point, a more regular and gradual decrease is observed. This suggests that the refraction has a more ductile character.

The areas under the force-displacement curves give amount of energy absorption. According to the updated analysis based on numerical integration of the curves, DAP resin-filled capsules 29.56 ± 1.42 N.mm; curing agent-filled capsules 20.4 ± 2.01 N.mm; empty capsules provided an energy absorption of 11.36 ± 0.92 N.mm.

**Figure 11:**
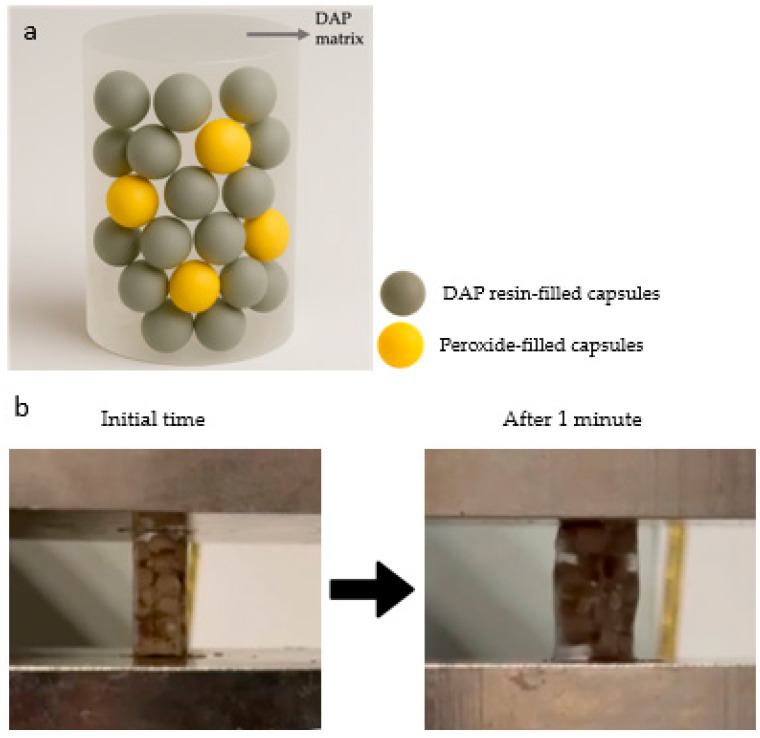
(**a**). Schematic representation of the compression specimen. (**b**) Change in material during compression testing.

The images of the specimens prepared according to the compression test standards before and after the test are shown in Figure 11.

**Figure 12:**
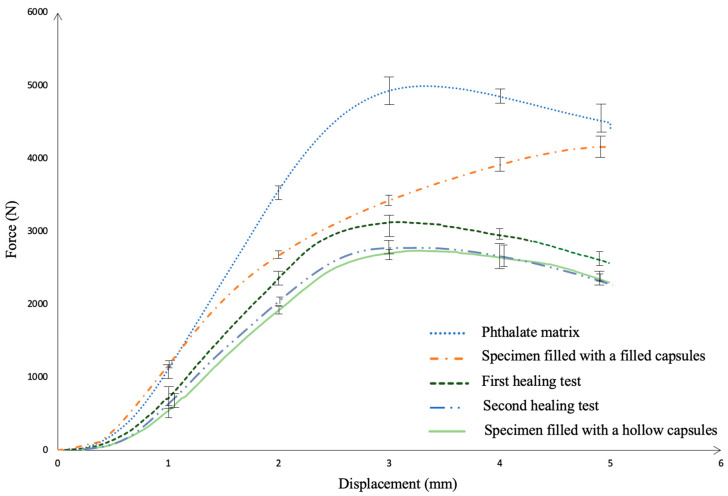
Compression test specimens using DAP resin.

The virgin DAP matrix has ensured the highest strength and can be taken as a reference. The post-healing specimen has successfully demonstrated the repair capacity of the capsules. The filled capsules and hollow capsules added composites showed lower performance than the virgin DAP specimen in terms of compression strength. The effectiveness of capsules within the matrix can be increased by optimization of the filling material and capsule-matrix bond (Figure 12). In these compression tests using the DAP matrix, 63.5% recovery efficiency was obtained at the end of the first healing, and 56% healing efficiency was obtained at the end of the second healing test.

**Figure 13:**
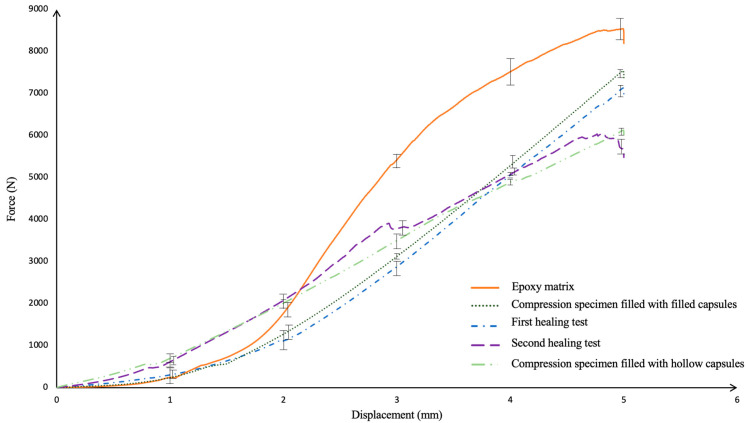
Compression test results of specimens created from epoxy matrix.

In Figure 13, the curve of the epoxy matrix shows a strength value of approximately 8620 N. Due to the homogeneous structure and high mechanical strength of epoxy, it has a higher load carrying capacity than other specimens. The structural integrity is high since there is no other material, such as a capsule inside. The DAP resin-filled matrix has reached a maximum force value of 7432 N. DAP resin-filled capsules showed an effect of increasing mechanical strength in the epoxy matrix but exhibited slightly lower strength than pure epoxy. DAP resin capsules have increased their strength by ensuring a homogeneous charge distribution within the matrix. However, DAP resin, which is a softer material than epoxy, can limit the strength of the matrix under high load. The test specimen with empty capsules shows a force value of 6212 N. It exhibited slightly lower strength compared to specimens containing DAP-filled capsules.

In the compression tests performed, it is seen that an increase in the strength of the material occurs as a result of the curing of the agents leaking from the filled capsules after healing. The healing agents infiltrating the cracks formed a flexible structure in the damaged areas.

Equation (1) (for the first healing test) and Equation (2) (for the second healing test) were used to calculate healing efficiency. The healing performance rate was 69% for the first healing test and 58% for the second healing test. In the first compression test to determine the recovery, the strength decreased compared to the initial epoxy and capsule combinations.

In the second compression test applied to the damaged specimen, the maximum force obtained decreased even further compared to the first test. The sudden drop in force, especially at a displacement of about 2.8 mm, indicated that although the healing agent had filled the previous damage zones, the microcracks that re-formed as a result of loading had started to propagate. This is due to stress concentrations focused on previously weakened areas, leading to sudden localized ruptures when a critical load threshold is exceeded. Furthermore, depletion of the healing fluid inside the capsules or loss of integrity of the capsule shell during the second healing cycle results in the inability to effectively repair repeated damage and localized stress concentration at these weak points. As a result, capsules only allow a limited number of re-healing cycles, and this effect must be taken into account in the material design.

## 4. Conclusions

In this study, the mechanical performance of self-healing materials was evaluated with the compression test results. The capsules used in the study were made of ceramic material resistant to heat and chemicals and filled with DAP (diallyl phthalate). The effectiveness of the self-healing mechanism, which is achieved by integrating the capsules into the material matrix, has been tested. The results revealed that the healing capsules positively affected material strength, and that the self-healing mechanism was feasible.

According to the compression test results, the DAP resin provided the highest mechanical strength and showed the best performance as reference material. The maximum force and deformation capacity is higher than all other specimens. The specimens tested after healing have largely recovered their initial mechanical strength, indicating that the capsules are effective in crack repair. This, thanks to the resistance of the ceramic capsules to heat and chemicals, ensured the successful release of the healing agent. Specimens reinforced with filled capsules showed higher mechanical strength than hollow capsules but did not perform as high as pure resin. This result shows that the interaction of the capsules with the matrix contributes to the load-carrying capacity. Specimens reinforced with hollow capsules offered the lowest mechanical strength but showed potential in terms of energy absorption or control of the fracture mechanism.

The curing efficiency of the capsules relies heavily on the reactivity, viscosity and quantity of the DAP liquid. Therefore, the shell–matrix interfacial interaction only plays a role as a secondary factor facilitating capsule release mechanics. In the DAP matrix, polar phthalate ester groups form secondary hydrogen bonds with -OH functions on the ceramic surface. The micro-roughened shell that develops with sintering provides mechanical interlocking and limits the displacement of the capsule. This makes it possible to store the reactive material. In the epoxy matrix, chemical bonding is not expected. However, the rough surface of the shell allows the epoxy to fill the micropores and creates a physical binding effect. The weak interface here promotes easier fracture of the capsules and release of DAP during crack propagation. Thus, in both matrix types, a balance is achieved that is robust enough to maintain capsule stability but compliant enough to allow fracture.

As a result, this study revealed the potential to improve the mechanical strength properties of self-healing materials using ceramic capsules. It has been observed that ceramic capsules resistant to heat and chemicals offer an effective crack repair and mechanical strength solution. It is considered that such materials can offer significant advantages in terms of sustainability, durability and cost-effectiveness in automotive, construction and other engineering applications. In future studies, it is proposed to examine parameters such as capsule content, capsule-matrix interactions, and long-term effects of the healing process in more detail.

## Data Availability

The data presented in this study are available on request from the corresponding author.
